# Chronic Thromboembolic Pulmonary Hypertension: the therapeutic assessment

**DOI:** 10.3389/fcvm.2024.1439411

**Published:** 2024-07-25

**Authors:** Beatrice Simeone, Enrico Maggio, Leonardo Schirone, Erica Rocco, Gianmarco Sarto, Luigi Spadafora, Marco Bernardi, Luca D’Ambrosio, Maurizio Forte, Daniele Vecchio, Valentina Valenti, Sebastiano Sciarretta, Carmine Dario Vizza

**Affiliations:** ^1^ Department of Cardiology, ICOT Istituto Marco Pasquali, Latina, Italy; ^2^ Department of Clinical Internal, Anesthesiological and Cardiovascular Sciences, Sapienza University of Rome, Rome, Italy; ^3^ Department of Angiocardioneurology, IRCCS Neuromed, Pozzilli, Italy; ^4^ Department of Medical-Surgical Sciences and Biotechnologies, Sapienza University of Rome, Latina, Italy; ^5^ Department of Cardiology, Santa Maria Goretti Hospital, Latina, Italy; ^6^ Department of Cardiovascular and Respiratory Sciences, Sapienza University of Rome, Rome, Italy

**Keywords:** narrative review, pulmonary hypertension (PAH), Chronic Thromboembolic Pulmonary Hypertension (CTEPH), therapy, rare disease

## Abstract

Chronic Thromboembolic Pulmonary Hypertension (CTEPH) is a severe and complex condition that evolves from unresolved pulmonary embolism, leading to fibrotic obstruction of pulmonary arteries, pulmonary hypertension, and potential right heart failure. The cornerstone of CTEPH management lies in a multifaceted therapeutic approach tailored to individual patient profiles, reflecting the disease's heterogeneity. This review delves into the current therapeutic strategies for CTEPH, including surgical pulmonary endarterectomy (PEA), balloon pulmonary angioplasty (BPA), and targeted pharmacological treatments such as PDE5 inhibitors, endothelin receptor antagonists, sGC stimulators, and prostanoids. Lifelong anticoagulation is also highlighted as a preventive strategy against recurrent thromboembolism. Special emphasis is placed on the interdisciplinary nature of CTEPH care, necessitating collaboration among PEA surgeons, BPA interventionists, PH specialists, and thoracic radiologists to ensure comprehensive treatment planning and execution. The review underscores the importance of selecting an appropriate treatment modality based on the patient's specific disease characteristics and the evolving landscape of CTEPH treatment, aiming to improve patient outcomes through integrated care strategies.

## 1 Introduction

Pulmonary hypertension (PH) is defined as a mean pulmonary artery pressure (PAP) greater than or equal to 20 mmHg as measured by right heart catheterization (RHC) (1, 2).

Chronic thromboembolic pulmonary (CTEPH), a potentially life-threatening condition, is defined as symptomatic pulmonary hypertension with persistent pulmonary perfusion defects despite adequate anticoagulation for 3–6 months (3) and represents a distinct disease entity classified as group 4 pulmonary hypertension (PH) according to ESC/ERS 2022 guidelines (2).

CTEPH is a rare and underdiagnosed complication of acute pulmonary embolism (APE) (4). Some characteristics of the original PE are associated with the development of CTEPH. Most significantly, unprovoked PE, a diagnostic delay of >2 weeks, and right ventricle (RV) dysfunction at the time of PE were found to be independent predictors of CTEPH (5).

Furthermore, the precise incidence of CTEPH after a documented and correctly treated APE is still unknown.

The exact epidemiology of CTEPH is unknown; it is most probably largely underdiagnosed and therefore undertreated, and demonstrate how much patients can benefit from modern multi-modal treatment concepts in expert centers.

Chronic thromboembolic pulmonary disease (CTEPD), is a general term proposed in the recent ERS Statement on Chronic Thromboembolic Pulmonary Hypertension (CTEPH) (6) to characterize symptomatic patients who present mismatched perfusion defects on ventilation/perfusion (V/Q) lung scintigraphy and specific signs of chronic organized clots on computed tomography pulmonary angiography (CTPA), conventional pulmonary angiography (CPA) or magnetic resonance imaging (MRI) after at least three months of therapeutic anticoagulation. Some of these patients have no pulmonary hypertension (PH) at rest (2), while the majority present with PH at rest, corresponding to the definition of CTEPH (group 4 of the updated clinical classification of PH) (1).

The natural history of CTEPH is complex. The current understanding of the pathophysiology of CTEPH has recently evolved beyond simple pulmonary vascular disease caused by intravascular mechanical obstruction due to organized chronic fibrotic material from unresolved clots. In fact, it may be a much more complex condition involving proximal and more distal obstruction of the pulmonary arteries associated with remodeling of the muscular pulmonary arteries, capillaries, and venules (7).

Nowadays, it is well accepted that pulmonary vascular remodelling can lead to significant pulmonary microvasculopathy, which plays a role in the development of a progressive increase in pulmonary vascular resistance and consequentially to the onset of right heart dysfunction and symptomatic CTEPH (8, 9). The processes by which residual organized clots persist after an APE are not fully understood, even if several risk factors have been identified.

The risk factors for CTEPH appear to differ from those for APE, such as immobilization or recent surgery. The potential risk factors for CTEPH include certain chronic medical conditions (e.g., permanent intravascular devices, inflammatory bowel diseases, autoimmune disease, hypothyroidism, splenectomy, and malignancy), thrombophilia, and genetic predisposition.

Regarding diagnostics, echocardiography, CT, and ventilation-perfusion (V/Q) scans can be utilized to confirm the suspicion of PH. The presence of a V/Q mismatch in the setting of PH should prompt further evaluation using RHC and pulmonary angiography. Each imaging modality has its role; thus, comprehensive evaluation using multimodal imaging is crucial for the proper diagnosis and management of patients with CTEPH. The rarity of the disease, nonspecific symptoms and signs, and a lack of physicians' awareness of CTEPH (including when to suspect it and how to evaluate it) are barriers to a timely diagnosis (10).

Therefore, despite the presence of effective therapy [pulmonary endarterectomy (PEA)], many patients are diagnosed only at the late stage of the disease, when distal PA obstruction and microvasculopathy have already progressed. Patients with advanced disease are not eligible for PEA because the procedure can only treat proximal lesions.

Luckily, in parallel with the advances in the diagnostic and surgical techniques for patients with operable CTEPH, considerable therapeutic advances including targeted medical therapy and balloon pulmonary angioplasty (BPA) have also been introduced for those deemed to have inoperable CTEPH due to the distal location of the chronic thromboembolic obstruction, severity of the hemodynamic impairment, presence of severe comorbid conditions, or personal preference (11–14).

Thus, a high index of suspicion and timely diagnosis using multimodal imaging tools is mandatory for optimal patient outcomes.

The present review aims to increase awareness of this rare disease and outline more recent efforts to identify solutions for the treatment of different phenotypes of CTEPH.

## 2 Therapy

**Figure 1:**
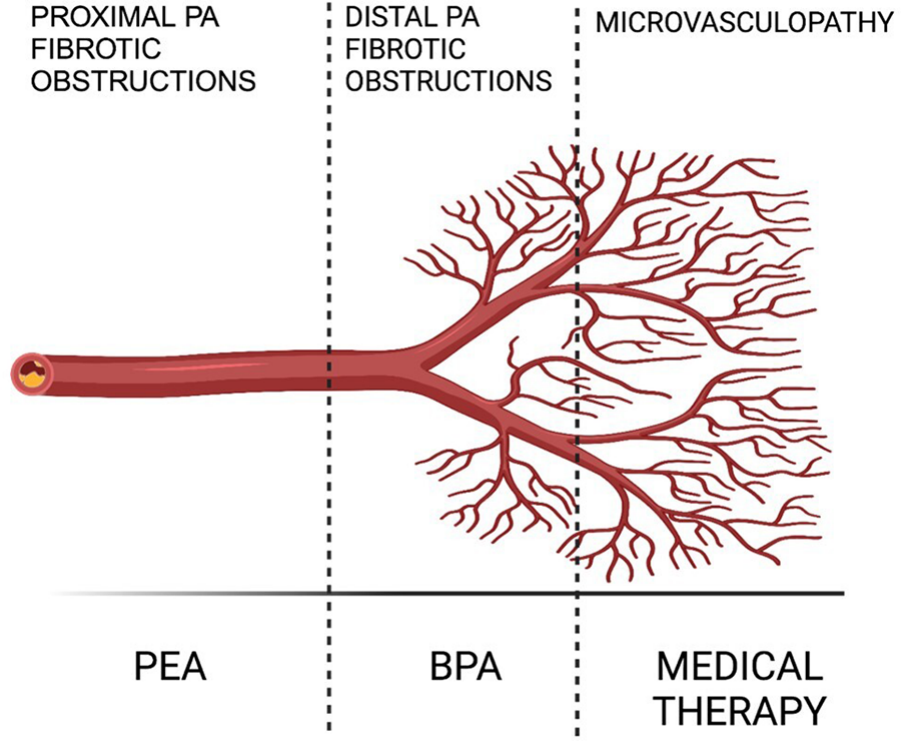
Multimodal CTEPH treatment (2).

The contemporary therapeutic strategy for Chronic Thromboembolic Pulmonary Hypertension (CTEPH) adopts a comprehensive, multimodal approach that integrates pulmonary endarterectomy (PEA), pulmonary balloon angioplasty (BPA), and pharmacological treatments to address the varied anatomical challenges presented by proximal, distal, and microvascular lesions (Figure 1). PEA stands as a potentially curative option, yet its applicability is limited by the surgical risk or inaccessibility of disease in certain patients, necessitating alternative interventions like BPA and medical therapy. In exceptional cases, like patients with mixed anatomical lesions (surgically accessible lesions in one lung and inoperable lesions in the other lung) might benefit from a combined approach with BPA (prior to or at the same time as surgery) and PEA to decrease the surgical risk and improve the final result (2).

### 2.1 Surgical treatment

Accurate diagnosis and meticulous patient selection are crucial for determining eligibility for pulmonary endarterectomy (PEA) in treating Chronic Thromboembolic Pulmonary Hypertension (CTEPH). Candidates for this surgical intervention should exhibit dyspnea on exertion classified at least as NYHA Functional Class II. The obstructive material must be identifiable on computed tomography (CT) scans and deemed surgically accessible, typically encompassing central, segmental, and subsegmental pulmonary arteries (15).

Indication for surgery in CTEPH patients depends on several factors: symptom severity, degree of PH and right heart dysfunction, level of stenoses and occlusions, correlation between PH and extent of obstructions, comorbidities and possible surgical difficulties, patient expectations and risk acceptance.

Recent findings suggest that PEA is feasible for a significant portion of CTEPH patients, particularly when decisions are informed by an interdisciplinary team evaluation (16, 17). Neither age nor comorbidities are considered absolute contraindications for PEA; however, potential risk factors affecting surgical outcomes and overall life expectancy must be carefully weighed in the decision-making process.

The criteria for selecting patients for PEA are detailed in Table 1 (18–20).

**Table 1:** Indication criteria for PEA.

1	Mean PA pressure ≥30 mmHg
2	Pulmonary vascular resistance ≥300 dyne·s·cm^−5^
3	NYHA/WHO functional class ≥III
4	The central end of the PA lesion must be in an area that can be reached surgically
5	No serious complications (comorbidities)

Although surgeons express concern when there is a very high PVR (especially >1,100 dynes) as this may imply more distal disease, current recommendations state that elevated PVR (>1,500 dyn·s·cm^−5^) alone is not a contraindication for surgery. In fact, there is no upper limit of PVR that makes a patient inoperable, provided there is a corresponding degree of obstructive disease. However, in some patients, a severely elevated PVR combined with other risk factors may render surgery unfeasible. Additionally, some patients with operable conditions may opt not to undergo surgery.

One key aspect is the technical accessibility of the obstructive material. This is mainly dependent on surgical expertise, which correlates with the case load of the individual surgeon. The surgical risk depends on the severity of hemodynamic impairment compared to the amount of obstructive material, comorbidities, and age of the patients (20).

Primarily, surgery is recommended for individuals with central-type CTEPH, characterized by obstructions in the main, interlobar, and segmental pulmonary arteries, accompanied by mural thrombi and intimal hyperplasia. Unfortunately, patients with peripheral CTEPH, where disease involvement extends to more distal pulmonary arteries, may not be suitable candidates for effective surgical intervention.

A significant surgical advancement in the treatment of Chronic Thromboembolic Pulmonary Hypertension (CTEPH) involves extending the distal limits of endarterectomy, allowing for successful surgeries even in cases of distal chronic thromboembolism at expert centers (20, 21). This progress is largely attributed to enhanced diagnostic capabilities and accumulated surgical expertise. Consequently, the University of California, San Diego has revised its previously published intra-operative classification to more accurately depict the contemporary surgical methodology and the extent of revascularization achieved (Table 2) (20, 22, 23).

**Table 2:** Pa occlusion morphology (University of California San Diego chronic thromboembolism surgical classification).

Surgical levels	Location of Chronic Thromboembolism (CTE)
Level 0	No evidence of thromboembolic disease in either lung
Level I	CTE starting in the main pulmonary arteries
Level Ic	Complete occlusion of one main PA with CTE
Level II	CTE starting at the level of lobar arteries or in the main descending pulmonary arteries
Level III	CTE starting at the level of the segmental arteries
Level IV	CTE starting at the level of the subsegmental arteries

Chronic thromboembolic disease (CTEPD) represents a unique subset of conditions closely resembling Chronic Thromboembolic Pulmonary Hypertension (CTEPH) in terms of symptoms and perfusion defects but distinctively lacks pulmonary hypertension (PH) at rest. This condition includes the same morphological signs of CTEPH, such as V/Q mismatches and clinical symptoms persisting despite anticoagulation therapy, without fulfilling the formal PH criteria [mean pulmonary artery pressure (mPAP) <20 mmHg]. In instances where a significant V/Q mismatch is present and symptomatic relief is anticipated, surgery may be considered. The decision to proceed with pulmonary endarterectomy (PEA) does not hinge solely on the degree of pulmonary vascular resistance (PVR) or right ventricular dysfunction, as neither is seen as an absolute contraindication. Surgical candidacy for PEA is determined on an individual and interdisciplinary basis, considering various factors outlined in specialized guidelines and should always be conducted at a center specializing in such treatments (Table 3) (15, 19).

**Table 3:** Summary of the main factors influencing the therapeutic indication for PEA.

1	Expression of the symptoms
2	Extent of hemodynamic changes: severity of PH, pulmonary vascular resistance and right ventricular dysfunction
3	Extent and localization of the morphological changes: lobar, segmental or subsegmental obstructive tissue
4	Correlation of morphological changes with the hemodynamics and the symptoms
5	Technical difficulties, previous operations, concomitant coronary artery or valvular diseases, other comorbidities with poor prognosis
6	Risk-benefit evaluation depending on the suffering pressure and the prognosis

Patients with contraindications or relative contraindications to cardiopulmonary bypass are approached with caution regarding Pulmonary Endarterectomy (PEA) since the procedure necessitates cardiopulmonary bypass through ascending aortic and caval cannulation, allowing for periods of deep hypothermic circulatory arrest (HCA). High-risk individuals or those with conditions such as neurological diseases, recent strokes, recent myocardial infarctions, bleeding disorders, severe left heart failure, active infections, other lung diseases, malignancies, recent pulmonary trauma, and multiorgan failure may not derive benefit from PEA (24).

PEA is performed via median sternotomy, connecting the patient to a heart-lung machine and cooling the body to 18°C–20°C to induce complete HCA. This state is essential to remove the obstructive thrombotic material within the intima and inner tunica media under optimal visibility, without collateral circulation backflow. Short periods of circulatory arrest, typically 20 min with interspersed reperfusion intervals, are employed to minimize the risk to the thin artery walls while ensuring thorough removal of the material into the distal vessels. Comorbid cardiac pathologies may also be addressed during this time, as coronary artery bypass graft (CABG) patent formane ovalis (PFO)/atrial septal defect (ASD) closure or partial anomalous pulmonary venous correction (19).

The procedure's complexity contributes to a broad spectrum of potential complications, including arrhythmias, postoperative right ventricular (RV) failure due to residual disease and persistent PH, reperfusion pulmonary edema and lung injury, ongoing hypoxemia, and risks of arteriotomy rupture or distal artery perforation leading to massive hemoptysis (25). Early complications like reperfusion pulmonary edema, manifesting as increased oxygen demand and pulmonary opacities, may necessitate prolonged intubation or VA-ECMO support. Right ventricular dysfunction could also lead to ECMO use, alongside other complications such as nosocomial pneumonias. Re-thrombosis in endarterectomized areas is rare, and while HCA may temporarily reduce vigilance, persistent cognitive dysfunction has not been linked to its use (17, 19, 26).

Untreated CTEPH has a 3-year survival rate of 70%, with variations based on hemodynamic severity (17, 27). PEA significantly improves prognosis, boasting a 3-year survival rate of 89% (17). Surgical mortality rates have drastically reduced from >20% to <5%, now comparable to other cardiac surgeries (26, 28, 29). Post-PEA, immediate hemodynamic improvements are seen, including reduced pulmonary artery pressure (PAP), with right ventricular remodeling and performance enhancement occurring over three to twelve months. One year post-PEA, significant reductions in pulmonary vascular resistance (PVR), improvements in the 6-min walk distance, and NYHA functional class improvements are observed. Persistent PH affects 17%–51% of patients, often due to microvascular disease, technical challenges in removing distal material, or comorbidities, yet these patients generally experience clinical benefits (20, 24).

### 2.2 Interventional treatment: balloon pulmonary angioplasty (BPA)

Pulmonary Balloon Angioplasty (BPA) is an interventional technique executed over multiple sessions, particularly beneficial for addressing distal subsegmental pulmonary artery obstructions that are not amenable to surgical intervention. In the short term, BPA has demonstrated superior impacts on hemodynamics, including improvements in the 6-min walking distance, pulmonary vascular resistance (PVR), and mean pulmonary artery pressure (mPAP), when compared to pharmacotherapy (15, 30). As such, BPA is typically reserved for patients with Chronic Thromboembolic Pulmonary Hypertension (CTEPH) who are not candidates for Pulmonary Endarterectomy (PEA) or those experiencing residual PH post-surgery (31). The primary indication for BPA is in scenarios where PEA is not an option and medication has proven ineffective, with specific criteria outlined for its performance (Table 4).

**Table 4:** Indications for BPA.

Difficulty in performing PEA	- Cases in which the lesion is below the regional artery, difficult to reach surgically, or proximal to the regional artery, but PEA is not performed due to complications that would interfere with surgery - Cases of residual or recurrent PH after PEA
Insufficient response to medical treatment	- NYHA/WHO functional class III or higher (mean PA pressure >30 mmHg or pulmonary vascular resistance >300 dyn·s·cm^−5^) despite drug therapy
Explanation and consent	- The patient (and family members) wishes to use BPA after having been fully informed of the medical condition and the risk-benefit ratio of BPA
Exclusion criteria	- Severe multiorgan failure, especially renal dysfunction

The potential of a sequential hybrid strategy combining PEA and BPA has been highlighted, with reports suggesting this approach may enhance outcomes (32). Additionally, studies have shown no significant difference in long-term survival between operated and non-operated CTEPH patients. This would suggest that a combination approach of PEA and BPA, rather than a single strategy, could lead to significant clinical improvements in the prognosis of CTEPH (33). Although further research is needed, there is an increasing body of evidence suggesting BPA's role extends beyond patients ineligible for PEA, potentially benefiting a broader cohort of CTEPH patients in conjunction with PEA or pharmacological treatments (34). BPA has been successful in alleviating symptoms, improving pulmonary function, exercise tolerance, and hemodynamic parameters (35–38). However, the specific clinical characteristics that predict improvement in exercise tolerance post-BPA remain to be fully elucidated.

A recent study has shed light on predictors of enhanced exercise tolerance following BPA, identifying young age, tall stature, short pre-procedure 6-min walk distance (6MWD), high mPAP, and high lung capacity (%VC) as factors associated with better post-BPA exercise outcomes. To accurately predict BPA's impact on exercise tolerance, a comprehensive pre-procedural evaluation encompassing physical activity levels, respiratory function, and PH severity is essential (39).

In hemodynamically and electrically stable patients without ongoing infections, if there are true absolute contraindications to right heart catheterization (RHC) — that is, conditions where the procedure would pose a significant risk to the patient that cannot be mitigated through pre-procedural optimization (such as severe coagulopathy, mechanical heart valves, severe hypoxemia, uncooperative patients, contraindications to sedation or anesthesia, or other severe comorbidities) — performing balloon pulmonary angioplasty (BPA) cannot be considered. Pre-treatment is necessary for those with iodine contrast allergies, a relative contraindication. Factors such as active infections, severe chronic obstructive pulmonary disease, hypoxemia, hematological disorders predisposing to bleeding or clotting, and uncontrolled systemic hypertension must be assessed before proceeding with BPA.

Preoperative optimization is crucial to minimize intraoperative and postoperative risks. Hemodynamic and systemic statuses should be stabilized, with preoperative oral vasodilators continued and inotropic support like dobutamine considered for severe cases with low cardiac output (40, 41).

BPA is conducted under conscious sedation, preferring femoral over internal jugular access. Target vessels, identified through perfusion imaging, CT angiography, and intra-procedural selective angiography, are approached via a long sheath through a short sheath inserted in the vascular segment of interest. Treating lower lobe vessels first, yet imaging and addressing all lung segments, is advised for comprehensive benefits. Anticoagulation during BPA aims for an activated clotting time of 200–250 s (40, 41).

Typically, 4–6 sessions are needed to address all lung segments and allow for remodeling, targeting the elimination of PH, prevention of right heart failure, and life expectancy improvement. Achieving a mean pulmonary arterial pressure of less than 25 mmHg and discontinuing home oxygen therapy by improving oxygenation are primary goals (40, 41).

The complication rate of BPA has significantly decreased, underscoring the importance of performing this procedure in experienced centers. Complications may arise from vascular injury due to wire perforation, balloon dilation, and high-pressure contrast injection (41).

Studies, including one in Japan and the RACE trial, have compared BPA's safety and efficacy to riociguat in inoperable CTEPH patients, showing BPA's superior improvement in mPAP and other hemodynamic measures despite a higher adverse event rate (42, 43).

BPA has also been shown to ameliorate metabolic and systemic disturbances associated with right ventricular dysfunction. Significant improvements in exercise capacity, muscle strength, WHO functional class, and potentially mental health-related quality of life post-rehabilitation post-BPA underscore its benefits (44–47).

In summary, while BPA is an alternative for patients ineligible for PEA, it does not supplant PEA but offers a potential choice for high-risk surgical candidates. As techniques advance, BPA could become preferred for certain patient groups, complemented by the prospect of hybrid procedures combining surgical and interventional treatments for comprehensive care.

### 2.3 Medical therapy

Medical therapy plays a critical role in the treatment of Chronic Thromboembolic Pulmonary Hypertension (CTEPH), particularly for addressing the microvascular disease component, as well as in patients deemed inoperable or those with persistent or recurrent PH following Pulmonary Endarterectomy (PEA). The foundation of drug therapy in CTEPH includes lifelong anticoagulation to prevent further thromboembolic events, alongside pulmonary vasodilators to manage PH. Diuretics may be prescribed to manage volume overload, and supplemental oxygen is used to correct hypoxemia when necessary.

Three significant clinical trials have made substantial contributions to the evidence base for the pharmacological management of CTEPH: CHEST-1 Trial (Riociguat), CTREPH Trial (Treprostinil) and MERIT-1 Trial (Macitentan), which will be discussed in greater detail later (Table 5).

**Table 5:** Main trials that studied different medications in CTEPH.

TRIAL	DRUG studied	Mechanism of the medication	Participants	Significant improvement in PVR	Significant improvement in 6-MWD
CHEST-1	RIOCIGUAT	Soluble Guanylate cyclase stimulator	261	YES	YES At 16 weeks
BENEFIT	BOSENTAN	Dual enothelin receptor agonist	157	YES	NO At 16 weeks
MERIT	MACITENTAN	Dual endothelin receptor agonist	80	YES	YES At 16 weeks
AMBER 1	AMBRISENTAN	Selective endothelin receptor agonist	33	Trend toward improvement in PVR and 6-MWD at 16 weeks	
SUNTHARALINGAM	SILDENAFIL	PDE-5 inhibitor	19	YES	NO At 16 weeks
CTREPH	TREPROSTINIL	Prostacyclin analog	105	YES	YES At 16 weeks
AIR TRIAL	ILOPROST	Prostacyclin analog	203	YES	YES

Drug therapy for CTEPH consists of anticoagulation therapy and pulmonary vasodilators including diuretics for volume overload and supplemental oxygen for hypoxemia when indicated.

#### 2.3.1 Anticoagulation

Lifetime anticoagulation therapy is a cornerstone in managing Chronic Thromboembolic Pulmonary Hypertension (CTEPH), crucial even after undergoing Pulmonary Endarterectomy (PEA) surgery. This recommendation is based on the disease's underlying pathophysiology, characterized by recurrent pulmonary thromboembolism and inadequate clot resolution. While the optimal anticoagulant choice remains undefined due to the absence of randomized controlled trials (RCTs) specific to CTEPH, warfarin has emerged as the expert-recommended and most commonly utilized agent. It is advised that warfarin be administered indefinitely, maintaining a Prothrombin Time-International Normalized Ratio (PT-INR) between 2.0 and 3.0 (48).

Recent years have seen an increase in the use of Direct Oral Anticoagulants (DOACs) as alternatives to warfarin, though their effectiveness in CTEPH has not been conclusively reported. A retrospective case series from the UK and the multicenter prospective registry (EXPERT) indicated similar bleeding rates between warfarin and DOACs among CTEPH patients. However, those on novel oral anticoagulants (NOACs) experienced higher rates of recurrent venous thromboembolism (49).

For patients with CTEPH and concurrent antiphospholipid syndrome—a condition affecting approximately 10% of the CTEPH population—Vitamin K Antagonist (VKA) therapy is deemed safer than DOACs, such as rivaroxaban (50). Therefore, in these cases, VKAs are recommended for anticoagulation (51).

Recent advancements in anticoagulation therapy for Chronic Thromboembolic Pulmonary Hypertension (CTEPH) are highlighted by two significant studies. The AXADIA-AFNET 8 trial suggests that apixaban offers a safe and effective alternative to vitamin K antagonists for patients with atrial fibrillation undergoing chronic hemodialysis, indicating potential benefits in a CTEPH context as well (52). The trial might provide valuable data on how to stratify risk in patients needing anticoagulation. This can help in developing strategies for identifying which CTEPH patients might benefit most from certain anticoagulants. Furthermore, understanding how different patient characteristics influence the effectiveness and safety of anticoagulation therapy in atrial fibrillation (AF), can help inform similar considerations in CTEPH patients, particularly since both populations can have overlapping comorbidities like hypertension and heart disease. Finally, it should be emphasized that theoutcomes regarding stroke, systemic embolism, and bleeding risks in AF patients can offer a comparative perspective on what to expect in a population that requires long-term anticoagulation, such as CTEPH. Meanwhile, the KABUKI trial, a multicenter, single-blind randomized study, found edoxaban to be non-inferior to warfarin in preventing the worsening of pulmonary vascular resistance in CTEPH patients, with a similar safety profile (53). These findings contribute to the evolving landscape of anticoagulation therapy in CTEPH, suggesting that direct oral anticoagulants like apixaban and edoxaban may offer viable alternatives to traditional therapy, warranting further investigation.

#### 2.3.2 Prostacyclin

Prostacyclin, a potent vasodilator and inhibitor of platelet aggregation and smooth muscle cell proliferation produced by endothelial cells, plays a significant role in the pulmonary vasculature's physiology. Its levels are found to be decreased in patients with Idiopathic Pulmonary Arterial Hypertension (IPAH), leading to the utilization of prostanoid medications in the treatment of Chronic Thromboembolic Pulmonary Hypertension (CTEPH) (54).

The CTREPH trial investigated the efficacy of treprostinil, a prostacyclin analog, in CTEPH patients who were either ineligible for Pulmonary Endarterectomy (PEA) or chose not to undergo the surgery. This study compared the effects of low-dose (3 ng/kg/min) vs. high-dose (30 ng/kg/min) subcutaneous treprostinil infusion over 24 weeks. The primary outcome, a change in the 6-min walk distance (6-MWD), demonstrated a significant improvement of +40 m with the higher dose of treprostinil, indicating its beneficial impact on exercise capacity (55). Consequently, treprostinil has been approved in the European Union for CTEPH treatment in patients classified as WHO Functional Class III–IV, either with inoperable disease or persistent/recurrent PH post-PEA, receiving a IIb B recommendation (2).

Additionally, long-term intravenous epoprostenol therapy has shown promising results in inoperable CTEPH patients. A study involving 27 patients revealed marked improvements in physical exercise capacity and Cardiac Index (CI) over a 20-month follow-up period. After 3 months of treatment, there was a notable improvement in NYHA Functional Class in 11 of 23 patients, a 6MWD increase of 66 m, and significant hemodynamic enhancements [mean Pulmonary Artery Pressure (mPAP), Cardiac Index, and Total Pulmonary Resistance], all indicating the therapy's effectiveness. Survival rates at 1, 2, and 3 years were 73%, 59%, and 41%, respectively, underscoring the potential life-extending benefits of epoprostenol in this patient population (56).

#### 2.3.3 Selexipag

Selexipag, an oral selective agonist for the prostacyclin receptor (IP receptor) with a non-prostanoid structure, and its metabolite MRE-269, exhibit high selectivity for the IP receptor, enhancing cyclic adenosine monophosphate (cAMP) production. This mechanism promotes vascular smooth muscle relaxation (57, 58). Previous studies in Pulmonary Arterial Hypertension (PAH) patients have shown selexipag's efficacy in reducing the risk of morbidity/mortality, resulting in its approval for PAH treatment in various regions, including the United States, European Union, and Japan (59).

In the context of Chronic Thromboembolic Pulmonary Hypertension (CTEPH), a phase 3, multicenter, double-blind, placebo-controlled study in Japan investigated selexipag's efficacy and safety for patients with inoperable CTEPH or persistent/recurrent PH post-PEA and/or BPA (60). The regimen began with 200 μg of selexipag twice daily, escalating up to 1,600 μg twice daily based on tolerability. Dose adjustments were made in 200 μg increments every three days to a total of six doses over 20 weeks, with the maximum tolerated dose maintained for the final 8 weeks.

The study primarily measured the impact on pulmonary vascular resistance (PVR), a critical hemodynamic parameter of PH that correlates with long-term prognosis. The reduction in PVR with selexipag aligns with outcomes from prior pulmonary vasodilator studies in CTEPH (12, 61, 62) and supports evidence from an earlier proof-of-concept trial hinting at selexipag's potential for hemodynamic improvement in Japanese CTEPH patients (63). Besides PVR, improvements were noted in other hemodynamic parameters, including PVR index, cardiac index, total pulmonary resistance, and mixed venous oxygen saturation (SvO2).

Despite these promising results, secondary endpoints such as the 6-min walk distance (6MWD) and WHO functional class did not show significant differences between selexipag and placebo groups, possibly due to the study's sample size.

The study concludes that selexipag is both well-tolerated and safe, offering hemodynamic benefits for CTEPH patients ineligible for PEA or those with ongoing PH after PEA and/or BPA, though no improvement was observed in exercise capacity. These findings underscore the need for further large-scale research to definitively establish selexipag's role in the management of CTEPH.

#### 2.3.4 Endothelin receptor antagonists

Endothelin-1, a potent vasoconstrictor and mitogen for smooth muscle cells produced by endothelial cells, has been found to have elevated plasma levels in patients with both Idiopathic Pulmonary Arterial Hypertension (IPAH) and Chronic Thromboembolic Pulmonary Hypertension (CTEPH) (64). Endothelin Receptor Antagonists (ERAs) mitigate the effects of endothelin-1 by either selectively blocking type A receptors or non-selectively blocking both type A and type B receptors, thus inhibiting the vasoconstrictive and mitogenic responses (65, 66).

The BENEFIT study, which assessed the dual endothelin receptor inhibitor bosentan in CTEPH patients, reached mixed outcomes. While bosentan effectively reduced Pulmonary Vascular Resistance (PVR), it did not significantly impact the 6-min walk distance (6MWD) over 16 weeks of treatment in 77 patients with symptomatic CTEPH who were either inoperable or had persistent PH more than 6 months post-PEA. The study also did not find a statistically significant difference in the time to clinical deterioration between bosentan-treated patients and the placebo group (80 patients) (11).

Reesnik et al. observed improved clinical and hemodynamic outcomes from bosentan treatment before endarterectomy. Yet, the limited number of trials conducted to date does not conclusively prove the benefit of this preoperative medical intervention (67).

Macitentan, developed as an improvement over bosentan with dual ETRA/ETRB antagonism, has been approved for the treatment of PAH. The phase 2 MERIT-1 trial, a double-blind, randomized, placebo-controlled study, evaluated the efficacy of 10 mg macitentan in 80 patients with inoperable CTEPH. After 16 weeks, the geometric mean PVR in the macitentan group decreased to 73.0% of baseline compared to 87.2% in the placebo group, indicating a significant improvement in PVR among patients with inoperable CTEPH (13). Furthermore, it is certainly important to highlight that 61% of patients enrolled in the MERIT study were taking a PDE5i, since this implies that the combination of Macitentan with a PDE5i may be beneficial in inoperable CTEPH.

Currently, a phase 3 RCT is underway to assess the safety and efficacy of 75 mg macitentan in patients with inoperable or persistent/recurrent CTEPH (NCT04271475), promising further insights into the therapeutic potential of macitentan in CTEPH management.

#### 2.3.5 Phosphodiesterase type-5 inhibitors

Nitric oxide (NO) is a critical endogenous vasodilator produced by endothelial cells, playing a pivotal role in inhibiting platelet aggregation and smooth muscle cell proliferation. NO activates soluble guanylate cyclase (sGC) to synthesize cyclic guanosine monophosphate (cGMP), leading to smooth muscle relaxation (12). Phosphodiesterase-5 (PDE5) inhibitors, like sildenafil, enhance vasodilation by preventing cGMP degradation. However, their effectiveness is contingent upon the presence of NO, which implies limited efficacy in lung regions with diminished NO levels (68, 69).

Sildenafil, specifically, has been studied for its impact on Chronic Thromboembolic Pulmonary Hypertension (CTEPH). In an open-label, uncontrolled trial by Reichenberger et al., 104 patients with inoperable CTEPH were treated with sildenafil at a dosage of 50 mg three times daily. The results showed a significant reduction in pulmonary vascular resistance (PVR) from a baseline of 863 ± 38 dyn·s·cm^−5^ to 759 ± 62 dyn·s·cm^−5^ after three months (*p* = 0.0002 vs. baseline), along with a notable increase in the 6-min walk distance (6MWD) from 310 ± 11 m at baseline to 361 ± 15 m at 3 months (*p* = 0.0001 vs. baseline) and 366 ± 18 m at 12 months (*p* = 0.0005 vs. baseline), indicating substantial hemodynamic and functional improvements (70).

While sildenafil's use in inoperable CTEPH has been off-label due to the absence of randomized controlled trials (RCTs) or registry data supporting its efficacy, oral combination therapy involving PDE5 inhibitors and endothelin receptor antagonists has become a prevalent approach for managing patients with CTEPH who exhibit severe hemodynamic compromise (71). This practice underscores the integration of various pharmacological strategies to alleviate the symptoms and improve the quality of life for those with this challenging condition.

#### 2.3.6 Soluble guanylate cyclase stimulator

Although numerous randomized controlled studies have assessed the efficacy and safety of various treatments, riociguat remains the only medication specifically approved by the FDA for both pulmonary hypertension (PH) and Chronic Thromboembolic Pulmonary Hypertension (CTEPH). Riociguat, a stimulator of soluble Guanylate Cyclase (sGC), is recommended for managing inoperable CTEPH and persistent/recurrent CTEPH after pulmonary endarterectomy (PEA), receiving IB recommendations (2, 72). This drug functions by directly stimulating sGC, enhancing cGMP levels independent of nitric oxide (NO) and increasing the sensitivity of vascular wall cells to endogenous NO, thus facilitating vasodilation in poorly ventilated lung areas (73).

Initial reports from 2010 by Ghofrani et al. demonstrated riociguat's ability to improve exercise capacity and reduce disease symptoms over a 12-week period in patients with CTEPH and PAH (74). The PATENT-1 study further validated riociguat's benefits in PAH patients, showing significant enhancements in exercise capacity, WHO functional class, pulmonary vascular resistance (PVR), and NT-proBNP levels (75). Long-term safety and efficacy were confirmed in the PATENT-2 trial, with sustained functional and exercise capacity improvements over one year (76).

The PATENT PLUS study explored the additive effects of riociguat with PDE5 inhibitors in PAH patients treated with sildenafil, finding no significant difference in hemodynamic improvements or exercise capacity between the groups (77). The CHEST-1 study among inoperable CTEPH patients reported similar improvements in exercise capacity, WHO functional class, PVR, and NT-proBNP levels with riociguat (12), with long-term benefits confirmed in the CHEST-2 trial (78).

However, despite these positive outcomes, the studies did not directly evaluate right heart dysfunction parameters, crucial for predicting survival. A comprehensive analysis by Marra A. et al., involving patients from the PATENT-1, PATENT PLUS, EAS, and CHEST trials, demonstrated significant reductions in right ventricular dimensions and improvements in TAPSE over 12 months of riociguat therapy (79).

It's important to note the absence of head-to-head trials comparing riociguat to other medical therapies for CTEPH, with differences in study designs making direct comparisons challenging. The CHEST-1, CTREPH, and MERIT-1 trials each had distinct patient populations and baseline therapy allowances, contributing to variability in patient demographics and disease severity (6, 12, 13, 54).

Riociguat stands as the sole FDA-approved treatment for inoperable CTEPH or post-PEA residual PH (72). While oral combination therapy, including PDE5 inhibitors and endothelin receptor antagonists, is prevalent in CTEPH patients with severe hemodynamic compromise (71), the role of vasodilator drugs as preoperative treatment remains controversial, potentially delaying surgery (67, 80). In contrast, for patients considered for BPA, pre-treatment with medical therapy may enhance pulmonary hemodynamics and procedural safety, although this recommendation is based on very low-quality evidence (2).

## 3 Therapeutic goals

The goal of CTEPH treatment is multifaceted, focusing on improving survival, symptoms, and quality of life, as well as hemodynamic parameters. Achieving a low-risk status in the European Society of Cardiology (ESC) risk assessment is associated with better outcomes. This involves parameters such as functional class, exercise capacity (6-min walk distance or cardiopulmonary exercise testing), biomarkers (BNP or NT-proBNP levels), and right ventricular function on echocardiography. A study by Hoeper et al. (81) demonstrated that patients who achieved or maintained a low-risk profile had significantly better survival rates compared to those in intermediate or high-risk categories.

Lowering the mean pulmonary arterial pressure (mPAP) is another important goal in CTEPH management. Elevated mPAP is a direct hemodynamic marker of disease severity and has been correlated with adverse outcomes. Surgical treatment, specifically pulmonary endarterectomy (PEA), is highly effective in significantly reducing mPAP and improving hemodynamics. Studies have shown that successful PEA can lead to normalization or near-normalization of mPAP, which is associated with improved survival and functional status (24). Additionally, the impact of mPAP on long-term outcomes has been highlighted in multiple studies. For instance, a study by Cannon et al. (82) found that post-operative mPAP is a strong predictor of survival in CTEPH patients.

Given that both achieving low-risk status and reducing mPAP are important, a comprehensive treatment strategy should aim at both. The ESC/ERS Guidelines for the diagnosis and treatment of pulmonary hypertension (2) recommend targeting a comprehensive low-risk profile, including mPAP reduction, functional improvement, and risk stratification parameters. Medical therapies, such as riociguat for inoperable or residual CTEPH, and interventional treatments, like balloon pulmonary angioplasty (BPA), also play a role in lowering mPAP and improving other risk parameters (68).

Currently, there's a lack of consensus on specific post-therapeutic targets for CTEPH patients post-PEA/BPA or medical therapy. Achieving a better WHO functional class (I-II), normalization or near-normalization of hemodynamic parameters at rest (as assessed by RHC 3–6 months post-procedure), and quality of life improvement are considered primary goals by experts (78).

Regarding the discontinuation of Pulmonary Arterial Hypertension (PAH) medications after achieving treatment goals with BPA or PEA, evidence suggests that it may be possible in certain cases. A study by Sugimura et al. indicated that after successful BPA, some patients could reduce or discontinue their PAH medications, particularly if they achieved substantial improvements in mPAP and other hemodynamic parameters (83). However, the decision to discontinue PAH medications should be individualized, taking into account the patient's overall stability and response to treatment.

In conclusion, the goal of CTEPH treatment should be to achieve and maintain a low-risk status as per the ESC risk assessment while also aiming to reduce mPAP. This dual approach is supported by evidence showing improved survival and quality of life with both strategies. Comprehensive management combining surgical, medical, and interventional therapies is essential to optimize outcomes in CTEPH patients.

## 4 Follow-up

To ensure the best outcomes for patients with Chronic Thromboembolic Pulmonary Hypertension (CTEPH), they must receive ongoing care at specialized centers dedicated to CTEPH management. These centers should feature a multidisciplinary team that includes a surgeon experienced in Pulmonary Endarterectomy (PEA), a Balloon Pulmonary Angioplasty (BPA) interventionist, a Pulmonary Hypertension (PH) specialist, and a thoracic radiologist, all of whom have training from high-volume PEA and/or BPA centers. Before discharge, patients must be thoroughly educated on the importance of medication adherence and the schedule for follow-up appointments. This is particularly vital for patients with multiple comorbidities, as diligent management of these additional health issues can markedly improve their functional capacity and overall quality of life.

**Figure 2:**
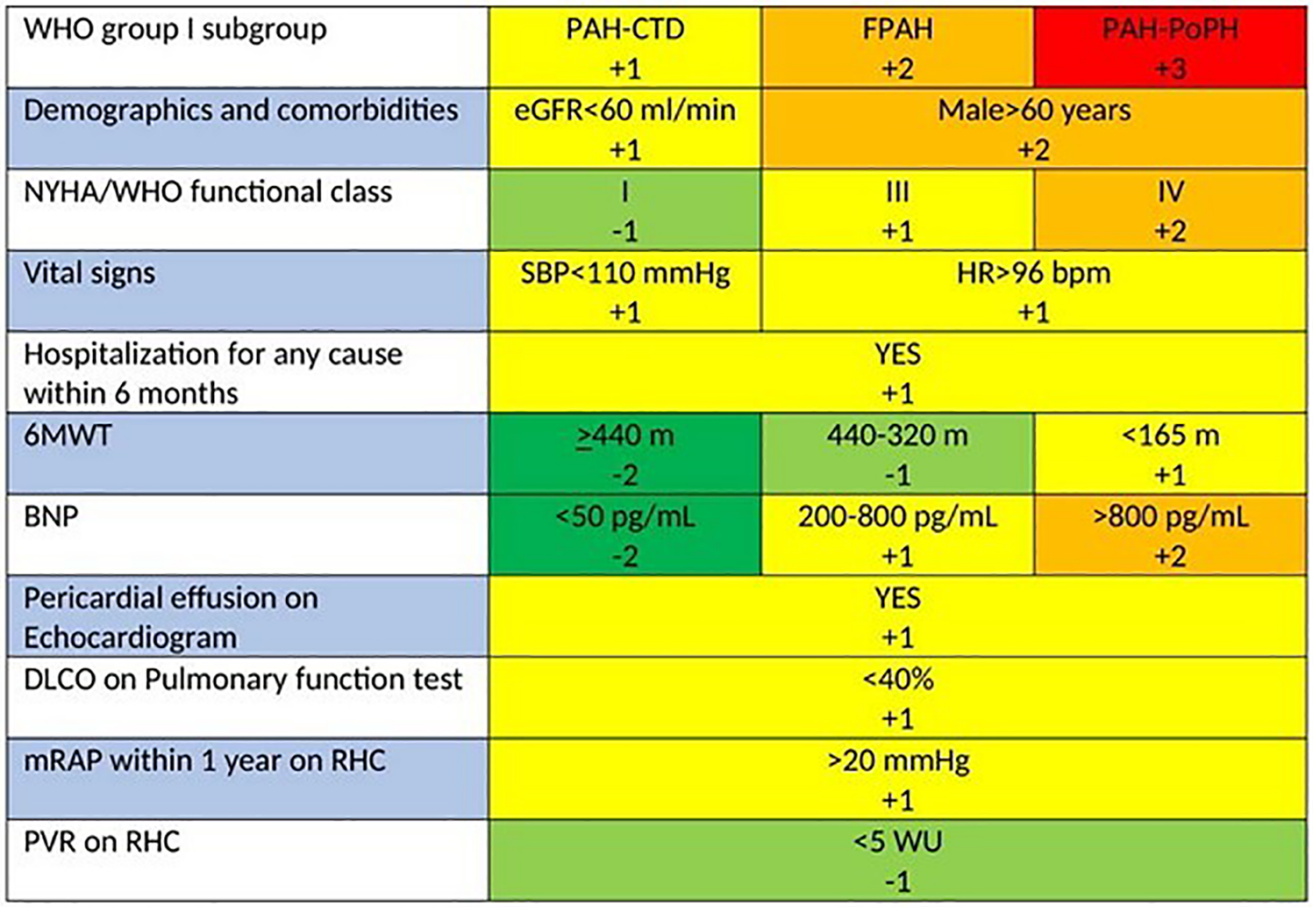
REVEAL 2.0 risk score calculator for PAH. Calculated risk scores can range from 0 (lowest risk) to 23 (highest risk). If N-terminal proBNP is available and BNP is not, listed cut-points are replaced with <300 pg/ml and ≥1,100 pg/ml (84).

While there is no specific recommendation for risk assessment in patients with inoperable Chronic Thromboembolic Pulmonary Hypertension (CTEPH), the REVEAL 2.0 risk score (RRS) provides a potentially useful tool (Figure 2). This updated version of the original REVEAL risk score calculator introduces revised variables and cut-off points, aiming to enhance risk prediction in Pulmonary Arterial Hypertension (PAH) and maintain clinical relevance. Its utility has been observed in differentiating prognosis regarding survival and clinical worsening-free survival (CWFS) or in predicting mortality among patients with inoperable or persistent/recurrent CTEPH (85, 86).

The RRS calculator integrates a composite, weighted risk algorithm with 13 evaluable elements deemed critical for patient outcomes. These elements encompass the 6-min walk test (6MWT), various hemodynamic parameters, renal function, and levels of N-terminal prohormone of brain natriuretic peptide (NT-proBNP). Despite its validation in PAH—a condition distinctly different from CTEPH—it was designed to forecast the likelihood of right heart failure, a significant determinant of prognosis and a common cause of mortality in both PAH and CTEPH (17, 87).

Created for a single-time risk assessment, the RRS calculator's application has expanded to allow serial evaluations. This adaptability enables healthcare providers to track a patient's risk profile and treatment response over time, offering a dynamic approach to managing individuals with PAH and potentially those with CTEPH as well (84).

**Figure 3:**
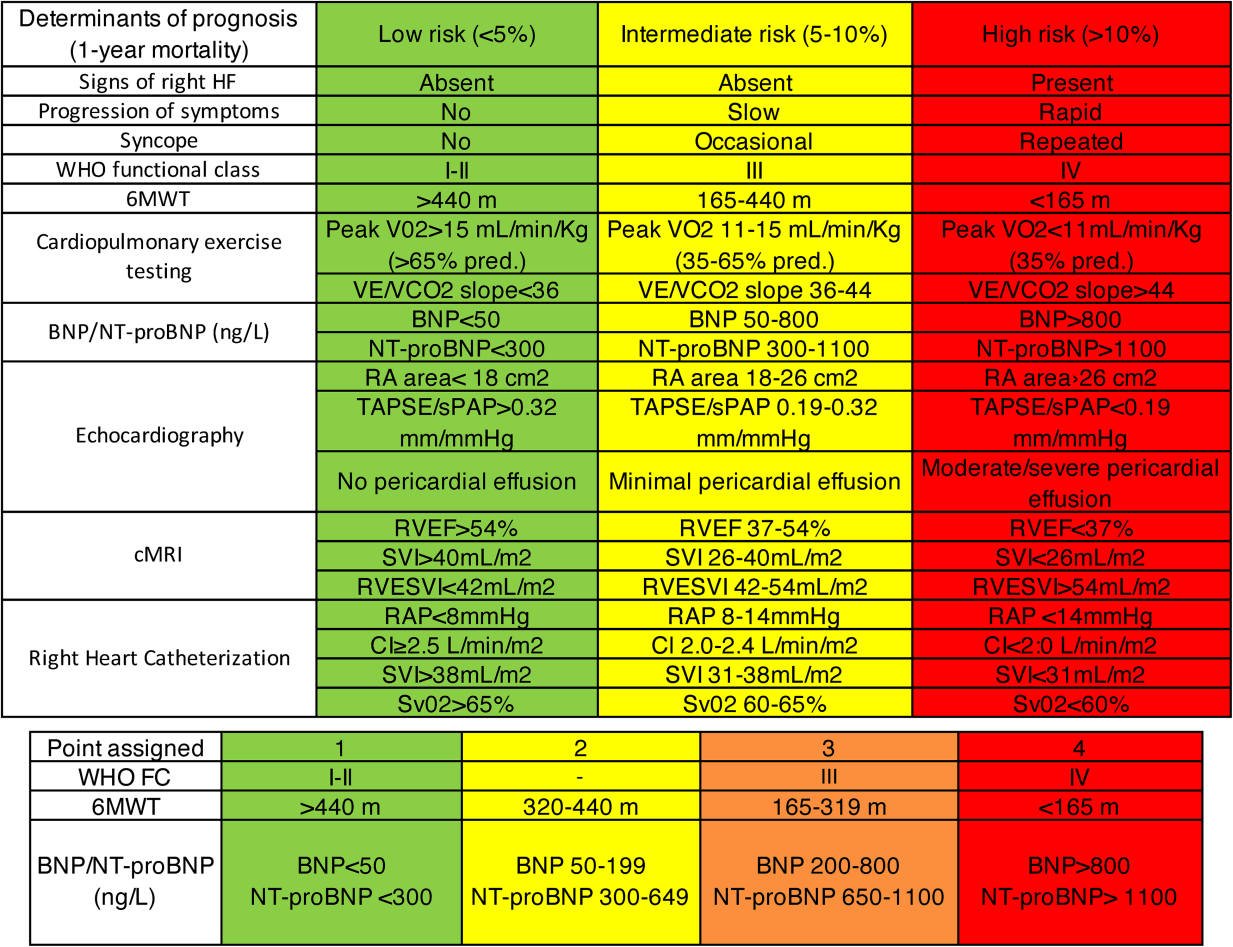
ERS/ESC 3- and 4-strata risk stratification (2).

**Figure 4:**
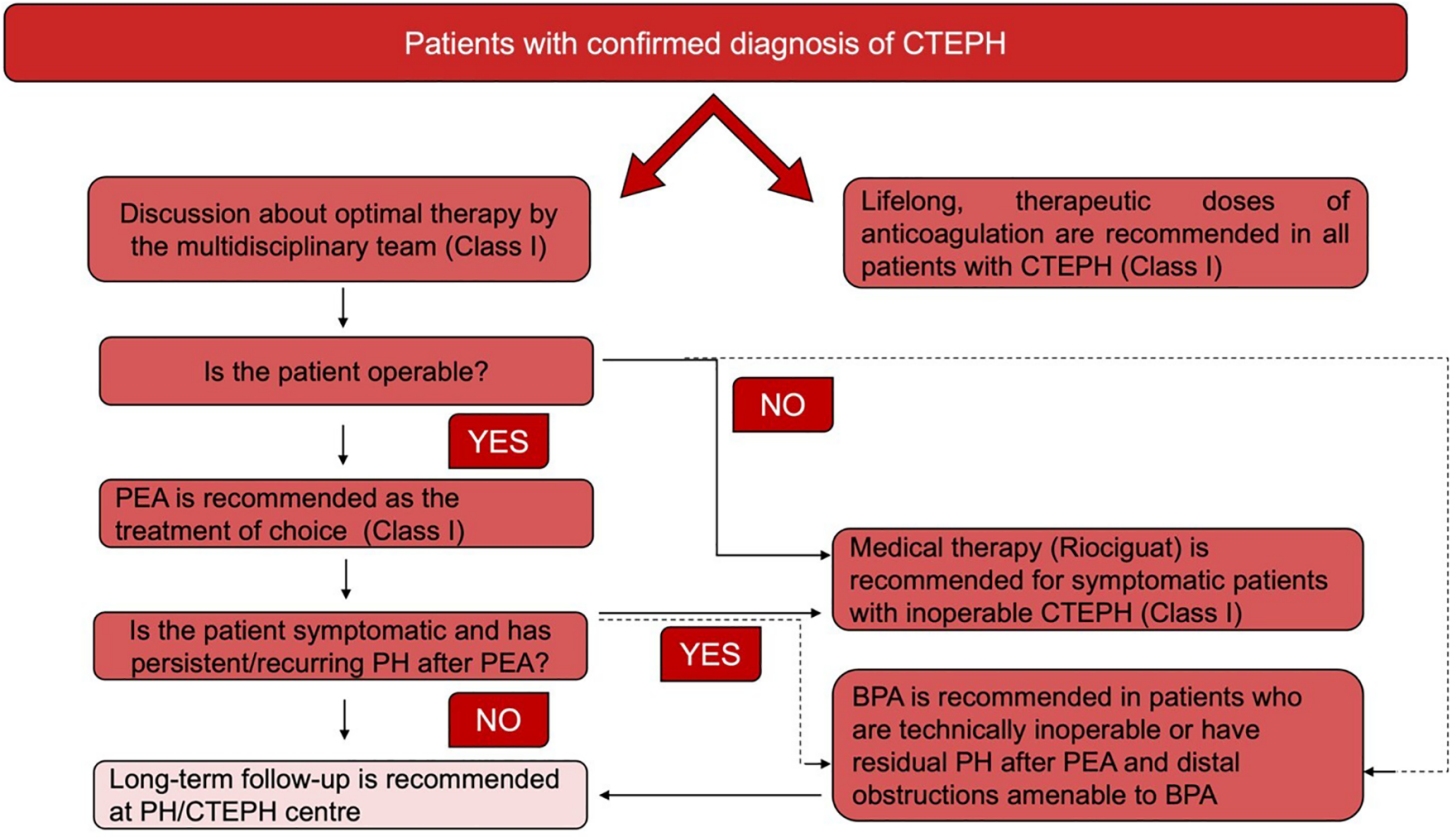
Management strategy in chronic thromboembolic pulmonary hypertension (2).

It's crucial to note that the ERS/ESC 3- and 4-strata risk stratification, intended for risk assessment at diagnosis and during follow-up, is another significant method (Figure 3). While this stratification has been validated for PAH, its application extends to CTEPH in clinical settings (2). This practice highlights the adaptability of the ERS/ESC risk stratification for use in managing CTEPH, despite its original validation for PAH (Figure 4).

Hemodynamic assessment through Right Heart Catheterization (RHC) at 3–6 months and 12 months after Pulmonary Endarterectomy (PEA) or Balloon Pulmonary Angioplasty (BPA) is crucial for stratifying the risk of mortality from Chronic Thromboembolic Pulmonary Hypertension (CTEPH) and determining the level of residual Pulmonary Hypertension (PH) to guide long-term management. Post-treatment, annual non-invasive follow-up involving transthoracic echocardiography (TTE) and cardiopulmonary exercise testing (CPET) is recommended due to the risk of recurrent PH (82).

Identifying patients with a low risk of residual PH could reduce unnecessary invasive follow-ups. This can be done using early postoperative hemodynamic data or non-invasive methods like TTE and CPET. Ruigrok et al. conducted an observational study to assess the efficacy and safety of utilizing early post-operative hemodynamics and 6-month follow-up data from NT-proBNP, CPET, and TTE to exclude residual PH. The study found CPET (peak V′O2 ≥80% predicted) to be effective in identifying patients with a low probability of residual PH, potentially reducing the need for repeat RHC without overlooking clinically relevant residual PH. However, larger cohort validation is necessary. It was also noted that CPET maintains its diagnostic value post-PEA, with TTE being a viable, albeit less predictive, alternative for excluding residual PH (88).

RV-PA coupling impairment is a key prognostic factor in PH. A retrospective study on CTEPH patients evaluated the prognostic ability of an echocardiographic measure of RV-PA coupling, the TAPSE/PASP ratio, in predicting adverse outcomes. This measure, combining changes in RV contractility and afterload, suggests that RV-PA uncoupling is significant in chronic PH, with the study's TAPSE/PASP ratios indicating severe uncoupling compared to acute PE. The TAPSE/PASP ratio, as a non-invasive marker, could thus reflect disease severity and predict outcomes, suggesting its potential incorporation into routine risk assessment and management of CTEPH (89, 90).

## 5 Conclusion

Treatment for CTEPH includes Pulmonary Endarterectomy (PEA), a surgical approach with curative intent, requiring significant surgical expertise and volume, and medical treatments like Balloon Pulmonary Angioplasty (BPA) for physically addressing PA stenosis or obstructions, and pharmacotherapy, including riociguat for those ineligibles for surgery.

PEA substantially improves functional and exercise capacity and hemodynamics, as well as life expectancy. Patients with distal, surgically inaccessible disease or residual pulmonary hypertension after surgery may substantially improve with medical therapy. BPA may be a promising option in patients with subsegmental disease alone or combination with surgery and, as all CTEPH treatment modalities, should only be performed at expert centers.

Despite advancements in understanding and managing CTEPH, many aspects of the disease remain unclear, necessitating ongoing research and clinical trials to unravel its pathogenic mechanisms and refine approaches for early detection and management.

The advent of multimodal therapy combining PEA, BPA, and pharmacological treatments marks a new era in CTEPH care.

This review aims to complete the training of clinicians by providing the fundamental treatment options and follow-up strategies for patients suspected of having CTEPH.

In summary, managing CTEPH demands a cohesive, multidisciplinary team approach for diagnosis, treatment implementation, and long-term patient monitoring, underscoring the need for specialized CTEPH teams to navigate the complexities of this challenging disease.
